# Impact of physical activity on ethoxy- and propoxypropanol human toxicokinetics in vivo

**DOI:** 10.1007/s00204-025-04094-0

**Published:** 2025-06-05

**Authors:** Hélène P. De Luca, Myriam Borgatta, Pascal Wild, Nicolas Concha Lozano, Gregory Plateel, Nancy B. Hopf

**Affiliations:** 1 Unisanté, University Center for Primary Care and Public Health & University of Lausanne, Lausanne, Switzerland; 2 Swiss Centre for Applied Human Toxicology (SCAHT), Missionsstrasse 64, 4055 Basel, Switzerland; 3 University Center of Legal Medicine (CUMRL), Universities of Lausanne and Geneva, Lausanne, Switzerland

**Keywords:** Propylene glycol ethyl ether, Propylene glycol propyl ether, Chemical exposure, Absorption, Elimination, Activity

## Abstract

Organic solvents such as propylene glycol ethers (PGEs) represents more than 20 different substances and are incorporated in thousands of commercial and professional products. Two PGEs commonly used in Europe and found mainly in cleaning and water-based paint products are propylene glycol ethyl ether (PGEE) and propylene glycol propyl ether (PGPE). Given their volatile properties, inhalation is the most common route of exposure. The aim of this study was to characterize human toxicokinetics following PGEE and PGPE inhalation exposure. The participants were exposed (4 h) at rest to a single PGE (between 15 and 35 ppm) under controlled conditions and blood, urine, and exhaled breath were collected. Our study shows that both PGEs were rapidly detected in blood (absorption rate: 0.01 µg/mL/h*ppm) and elimination was more important through urine (half-life: 1 h) than exhaled breath (half-life: 2 min). We also evaluated the impact of a moderate physical activity (30 min, 100 W) during exposure. A significant increase of blood absorption (absorption rate: 0.03 µg/mL/h*ppm) and internal dose (increase of 48%) was observed. Our results confirm that both PGEs are easily absorbed at rest and even faster with a moderate physical activity. The biomonitoring approach we have developed here allow the measurement of the “real” internal dose in workers handling these solvents. The existing occupational exposure limits do not consider workload, which may lead to their underestimation. Therefore, we recommend the use of biomonitoring for future studies and the consideration of physical workload for future exposure limits settings as an important parameter for risk assessment.

## Introduction

Propylene glycol ethers (PGEs) represent more than 20 chemicals with different molecular structures found in thousands of products for both consumers and professionals. These chemicals are organic solvents and part of the glycol ether family. Their amphiphilic and solvent properties make them useful in paints, cleaning products, inks, and cosmetics (Cicolella 2006). This new family of molecules first appeared on the market in the 1990 s (Cicolella 2006; Multigner et al. 2005). Some of the most present PGEs on the European and Swiss market are propylene glycol ethyl ethers (PGEE) and propylene glycol propyl ethers (PGPE). Both are labeled as eye and skin irritants, and PGEE is also categorized as inducing central nervous system toxicity (European Chemical Agency 2022a, 2022b). Because of their volatile properties, the most common route of exposure is inhalation, however, exposure through ingestion and skin absorption is also possible (Fiserova-Bergerova 1985; Sainio 2015). Workers are often the most exposed population because exposures are often daily and to concentrations that can reach the occupational exposure limits (OEL). These are set to limit possible negative health effects due to chemical exposure. OELs indicate maximum levels of exposure that are considered to be safe for worker exposed 42 h a week, 8 h a day, over many years. Some countries have derived OEL values for PGEE; Switzerland with 50 ppm (or 220 mg/m^3^) (SUVA 2024) and Germany 86 mg/m^3^ (MAK Commission 2023). No OEL value exists for PGPE. The toxicological and epidemiological results are necessary to set the OELs and determine the critical points for health (National Academies of Sciences 2019). Toxicokinetics provide important data on rates of absorption, distribution, metabolization, and elimination (ADME) of a chemical (Borgatta et al. 2022), However, toxicological, and epidemiological studies on PGEs are scarce. No such studies exist for PGPE, which partially explains the absence of an OEL for this chemical. Setting such limits can protect hundreds of thousands of workers that are exposed every day.

Human toxicokinetic studies are usually performed at rest conditions where participants are seated on a chair throughout the exposure session. However, physical activity should be considered when assessing exposures to cleaning products, paints or other products containing PGEs. Depending on their occupational filed and tasks, the workers may engage in physical activities leading to physiological changes. Pulmonary and alveolar ventilation increase with respiratory rate and tidal volume during physical activities (Aliverti 2016; Csanády and Filser 2001). An increased cardiac output also leads to a redistribution of blood flow where exercising muscles receive more blood, while the liver and the kidneys receive less (Joyner and Casey 2015; Volianitis and Secher 2016). Physical activity also impacts numerous other physiological factors affecting toxicokinetic, such as pH or the body temperature (Löf and Johanson 1998). These physiological changes can impact ADME by increasing or decreasing absorption and elimination of the chemical influencing the internal dose. Some studies highlighted the impact of a physical activity at different workload (effort in Watts, W) on toxicokinetic using physiologically based toxicokinetic (PBTK) models (Hamelin et al. 2005; Sari-Minodier et al. 2009) or performing controlled inhalation exposures with human participants (Göen et al. 2016; Nadeau et al. 2006; Tardif et al. 2007; Truchon et al. 2009). All studies reported a significant increase in chemical concentrations in the alveolar air as well as an increase in urinary metabolite concentrations with the increasing workload. Although these results show the impact of physical activity on chemicals absorption, human studies including exertion remain very rare. The impact of varying workloads and tasks on physiological and toxicokinetic processes is frequently ignored in chemical risk assessments. Such results are essential to avoid underestimating PGEs inhalation exposure during physical activities. Furthermore, the new data can be directly used to develop more precise OEL values to protect workers exposed daily to these chemicals.

The aim of this toxicological study was to provide human toxicokinetic data for inhalation of PGEE or PGPE at rest and with a moderate physical activity. Three different air concentrations of PGEs were used to provide an external-internal exposure dose relationship. Moreover, exposure sessions were performed at rest and with a moderate physical activity to highlight a possible impact of workload in PGEE and PGPE blood absorption and internal dose (*i.e.*, fraction of the chemical that reaches blood (Burcham 2013) as well as urinary and exhaled breath elimination.

## Methods

### Chemicals

PGEE (CAS no. 1569-02-4, > 95%) and PGPE (CAS no. 1569-01-3, > 99%) used in this study were obtained by Chemie Brunschwig AG (Basel, Switzerland). Propylene glycol butyl ether (PGBE, CAS no. 5131-66-8, ≥ 99%) used as internal standard was purchased from Sigma Aldrich (Buchs SG, Switzerland).

### Human participants

The participants were healthy according to a health questionnaire completed before entering the study and with blood parameters within the normal clinical ranges (sodium, kalium, red blood cells, hemoglobin, aspartate transaminase (AST), transaminase (ALT), γ-glutamyl-transferase (GGT), and creatinine concentrations). The participants included in our study had to be aged between 18 and 65 with a body mass index (BMI) between 18 and 25. Other inclusion criteria were the following: non-smoker, no daily alcohol consumption, not under medical regime, and not occupationally exposed to glycol ethers. Women were not under hormonal contraception regime, not breastfeeding, and not pregnant. A pregnancy test was always completed for all non-menopausal women before the first exposure session.

### Experimental design

**Fig. 1:**
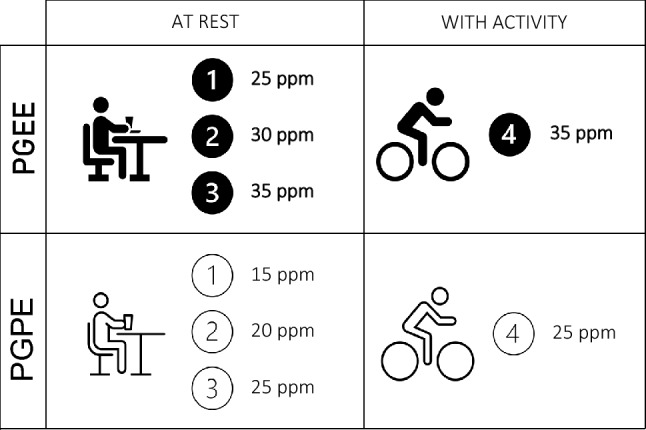
Exposure design for participants exposed to PGEE or PGPE vapors at rest (including three different air concentrations) and with physical activity

All exposure sessions were performed under controlled conditions at the Department of Occupational and Environmental Health, Center for Primary Care and Public Health (Unisanté), University of Lausanne, in Switzerland. PGEE or PGPE air concentrations were generated in a 12 m^3^ exposure chamber (Guillemin 1975) and monitored electronically (Lab VIEW software, National Instruments Corporation, Texas, USA) with a portable infrared spectroscopy gas analyzer for ambient air analysis (GASMET™ analyzer DX-4040 Gasmet Oy, Helsinki technologies, Finland) (Devanthery et al. 2003; Tomicic et al. 2011). During each exposure session, the participant spent 4 h inside the exposure chamber, and then 2 h outside in a solvent-free area. Each participant took part in four different exposure sessions comprising four different experimental conditions. Three sessions were performed at rest at either 25, 30, and 35 ppm of PGEE or 15, 20, and 25 ppm of PGPE. The fourth session was performed with moderate physical activity at 35 ppm of PGEE or 25 ppm of PGPE (Fig. 1).

The air concentrations were below the existing OEL for PGEE, *i.e.*, 50 ppm. Given the absence of an OEL for PGPE, the chosen concentrations were previously tested to ensure odor tolerance for participants. The participants performed the physical activity at 1.5 W/kg on an ergonomic bicycle (Corival, Lode B.V., Groningen, The Netherlands) for 30 min twice during the same exposure session: the first without solvent exposure and the second with PGEE or PGPE. The physical activity design was adapted after discussion with sports science researchers from the University of Lausanne. A minimum of 48 h between two exposure sessions was determined to avoid possible cumulative exposure (Borgatta et al. 2022). Blood, urine, and exhaled breath samples were collected before, during, and after all exposure sessions. The timing of sample collection was based on previous studies and protocol testing.

#### Blood collection

The blood samples were collected with an intravenous catheter inserted in the participant’s forearm by a research nurse into glass tubes (Vacutainers® NH Heparine Sodium, Becton Dickinson AG, Basel, Switzerland). A first blood sample was collected before the exposure session in a solvent-free area and used as baseline value. The timing following the first blood sample was 30, 120, 180, 240, 255, 300, and 360 min after the exposure to PGE started. The last blood sample was collected 24 h post-exposure. The blood samples were kept in the dark at 4 °C until analysis.

#### Urine collection

The participants were asked to provide a urine sample before the exposure session (solvent-free area, baseline) and then, every 2 h over 6 h. The participants continued to collect their urine (*ad lib)* samples at home for additional 24 h and bring them back in a cooler with cooling elements the day after the exposure session. They provided urine collection date, time, and total urinary volume. All urine samples were stored in polypropylene cups in the dark at 4 °C (Borgatta et al. 2021). The cold storage is important to avoid any loss of substance as glycol ethers are volatile organic compounds.

#### Exhaled breath collection

The exhaled breath samples were collected using a non-invasive simple-to-use device for biological exposure monitoring of volatile organic compounds (BioVOC-2 Breath Sampler, Brechbühler, Schlieren, Switzerland). The last portion of expired breath (129 mL) was collected on thermal desorption (TD) tubes (Tenax® TA, Camsco, Böckten, Switzerland) from each participant. A first exhaled breath sample was collected before the exposure session in a solvent-free area (baseline). The timing following the first exhaled breath sample was 120, 240, 255, 260, 265, 270, 275 and 360 min after exposure had started and a last one 24 h post exposure. Exhaled breath samples were analyzed immediately after collection.

### Chemical analysis

Gas-chromatography (Trace 1310, Thermo Scientific, USA) coupled with mass spectrometry (GC–MS/MS) (Thermo Scientific TSQ 8000 Evo, USA) equipped with a solid phase microextraction fiber (SPME), a capillary column (ZB FFAP 20 m, 0.18 mm, Phenomenex, USA), and an autosampler (TriPlus RSH, Thermo Scientific, USA) was used to quantify glycol ethers in blood and urine. To quantify free form of the compound (not conjugated), blood samples were diluted (1:1) with deionized water (MilliQ, Merck Millipore, France) and 1 g of sodium sulfate (Na_2_SO_4_, CAS no. 7757-82-6, Sigma-Aldrich, USA) was added to urine samples. To quantify total form of PGEE or PGPE (the sum of conjugated and free parent compound), diluted blood and urine samples were incubated with 200 µl HCl (Hydrochloric acid 30%, Suprapur, CAS no. 7647-01-0, Merck KgaA, Germany) at 38 °C for 2 h. Once at room temperature, 200 µl NaOH (sodium hydroxide 10 M, CAS no. 1310-73-2, Merck KgaA, Germany) was quickly added to neutralize the HCl. The headspace (HS) glass vials (20 mL) with the diluted solutions and the internal standard (PGBE, 1.5 µg/sample) were incubated at 90 °C (10 min). The fiber (Carboxen/PDMS, 85 um thickness, Supelco, Bellefonte, USA) extracted for 1.5 min at 90 °C and the samples were desorbed in the GC injector at 280 °C for 8 min. The GC program was 40 °C (1 min), increased to 125 °C at 6 °C/min (total cycle time was 15 min). The limits of quantifications (LOQ) were 0.04 µg/mL PGPE and PGEE.

Thermal desorption–gas chromatography–mass spectrometry (TD-GC–MS, GC column (Agilent VF-624), Thermo Trace 1310, ISQ LT) was used to quantify glycol ethers in exhaled breath. The absorbent tubes (TENAX^®^ TA, Camsco, Böckten, Switzerland) were desorbed at 300 °C for 7 min (flow rate 40 ml/min) before injected into the TD-GC–MS for analysis. The LOQ was 40 ng/tube for both solvents.

### Statistical analysis

The maximal concentration (Cmax) of PGEs within each exposure session as well as the time this maximal concentration was reached (Tmax) were computed for each participant for blood, urine, and exhaled air. The absorption of PGEE and PGPE in each biological media (blood, urine, and exhaled air) from t_0_ until Cmax was modeled using a linear mixed model. The fixed independent variables for all exposure sessions were the cumulative dose computed as the time exposed multiplied by the air concentration of exposure with participant-specific random effects as well as participant-specific random slopes. The elimination of PGEs in all biological media from Tmax (or the end of exposure whichever was earlier) to the last measurement was modeled using a linear mixed effect model of the log-transformed concentration. The independent variable was the time (in minutes) since Tmax and with participant-specific random effect as well as participant-specific random slope. The half-life of excretion was computed as log(2)/elimination slope with the corresponding confidence interval computed from the confidence interval of the slope. The area under the curve (AUC, µg*min/mL) was calculated as follows:

1 $$\Delta {AUC}_{<span class='convertEndash'>1-2</span>}=\frac{{C}_{{y}_{1}}+{C}_{{y}_{2}}}{2}\times \left({t}_{{x}_{2}}-{t}_{{x}_{1}}\right)$$

where C [µg/mL] is the concentration measured in the biological samples and t [min] represents the time in minutes (Borgatta et al. 2022). The percentage of PGE eliminated through urine and exhaled air was calculated dividing the corresponding AUC by the AUC of blood.

## Results

In this study, the concentrations of free (not conjugated) and total PGEs (the sum of conjugated and free parent compound) were very close for both molecules, so we presented only the results of free PGEs. A total of four women and five men were recruited in our study with a mean age of 23.9 ± 2.4 years old and a mean BMI of 22.4 ± 2.2. The following sections present first the results for exposure sessions performed at rest, followed by the comparison of the results obtained during the physical activity.

### Blood absorption of PGEs during exposure sessions at rest

**Fig. 2:**
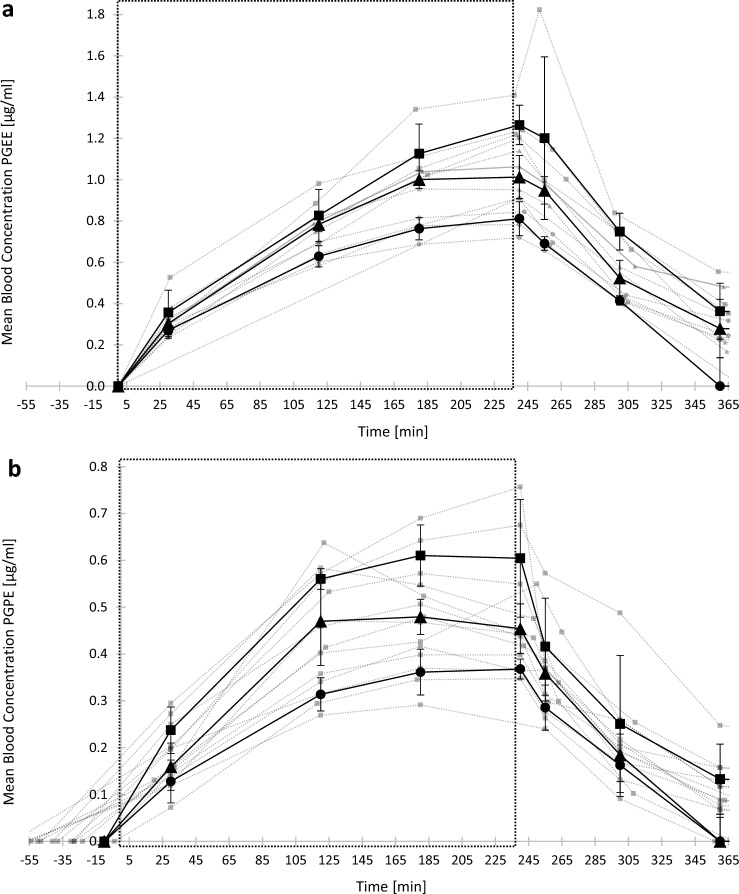
External-internal exposure dose relationship curves in blood (µg(mL) over the time (minutes) following four hours inhalation exposure to PGEE or PGPE at rest. Each grey curve represents the data from one participant. Black curves are the average (± standard deviation) for all participants (*n* = 5). The large square represents the time in the exposure chamber. **a** PGEE concentrations in blood as a function of time for a 25 (●), 30 (▲) and a 35 (■) ppm exposure to PGEE vapors. **b** PGPE concentrations in blood as a function of time for a 15 (●), 20 (▲) and a 25 (■) ppm exposure to PGPE vapors

Figure 2 shows the geometric mean PGEE (Fig. 2A) and PGPE (Fig. 2B) blood concentrations over time. The blood concentrations of PGEE and PGPE were always under the LOQ before exposure for all participants. The Cmax following PGEE exposure at rest was reached after four hours (240 min) of exposure for all air concentrations, *i.e.*, at the end of exposure. The Cmax following PGPE exposure at rest was reached for most of the participants after three hours (180 min). For a same concentration of exposure, the average Cmax in blood were 0.81 ± 0.08 and 0.65 ± 0.09 µg/mL following PGEE and PGPE, respectively. The results showed a linear blood absorption and an exponential blood elimination rate for both solvents. PGEs blood concentrations were back to baseline the day after the exposure session. The half-life of PGEE and PGPE in blood was 60 and 54 min, respectively.

### Blood absorption of PGEs during exposure sessions with physical activit

**Fig. 3:**
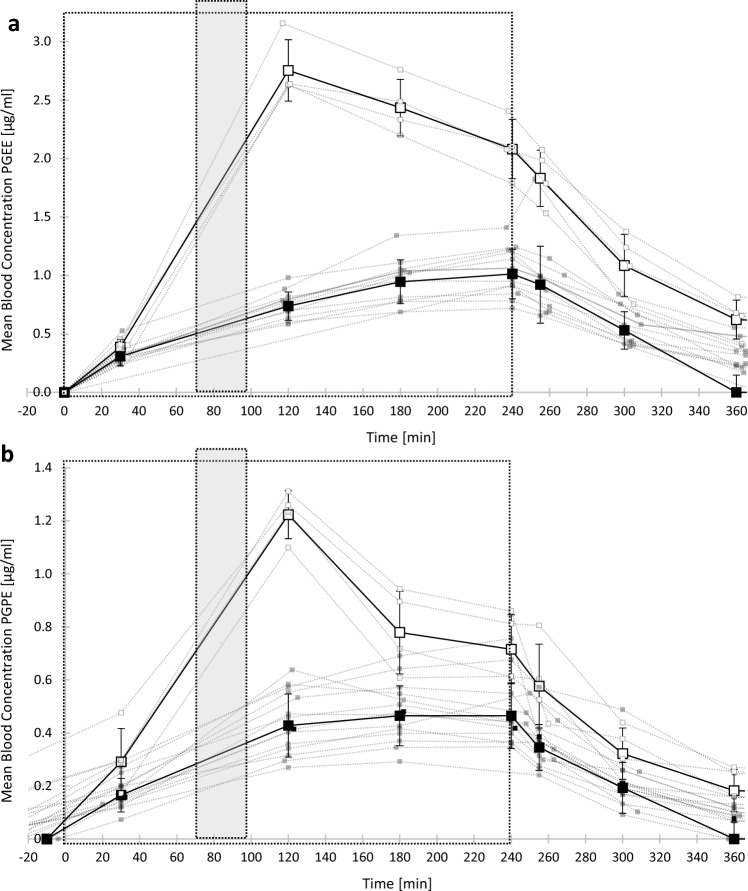
External-internal exposure dose relationship curves in blood (µg(mL) over the time (minutes) following exposure at rest (■) or with physical activity (□). Each dotted line represents the data of one participant. Bold points are the average (± standard deviation) for all participants (*n* = 9). The large square represents the time in the exposure chamber (4 h). The grey rectangle represents the 30 min of physical activity. **a** Exposure sessions with 35 ppm of PGEE. **b** Exposure sessions with 25 ppm of PGPE

Figure 3 shows the geometric means of PGEE (Fig. 3A) and PGPE (Fig. 3B) quantified in blood during exposure sessions with physical activity. The results at rest are also provided for a clear comparison. In exposure sessions at rest, the absorption rates during the first 2 h of exposure were similar through all air PGEs concentrations. Thus, in this figure, the external-internal exposure dose relationship curve at rest is the average of all external-internal exposure dose relationship curves presented in Fig. 2. For sessions with physical activity, Cmax was reached after 2 h (120 min) after the beginning of exposure, i.e., at the end of the physical activity. PGEs blood concentrations were back to baseline the day after all exposure sessions.

Table 1 summarizes the geometric means and the standard deviations as well as the 95% confidence intervals of Cmax and absorption rates for both solvents exposure at rest and coupled with physical activity. The results show a significant increase (2.5 times higher) for Cmax in blood for the first 2 h of exposure for all exposed participants (PGEE: *p* = 0.009; PGPE: *p* = 0.0002) as well as a significant increase for the blood absorption rate (PGEE: *p* = 3.93*10^–31^; PGPE: *p* = 2.57*10^–26^) for both solvents (Table 1). There is also a significant increase (1.5 times greater) in PGEE urinary absorption rate (*p* = 0.009) for the first 2 h of exposure but did not differ significantly (*p* = 0.09) with physical activity following PGPE exposure (Table 1). The results did not show significant difference either in Cmax in exhaled breath (PGEE: *p* = 0.38; PGPE: *p* = 0.45) or in urine (PGEE: *p* = 0.1; PGPE: *p* = 0.08), nor in absorption rate in exhaled breath (PGEE: *p* = 0.41; PGPE: *p* = 0.61) between resting and activity exposure conditions following PGEs exposure (Table 1).

**Table 1:** Absorption kinetic parameters (geometric mean ± standard deviation) with 95% confidence intervals for PGEE and PGPE in blood and urine, in resting conditions or coupled with physical activity. Area under the curve (AUC), maximal concentration (Cmax), and absorption rate (Absorption)

Parameters	Concentration of PGEE				Concentration of PGPE			
	Blood		Urine		Blood		Urine	
At rest								
AUC [µg*min/mL]	438.96 ± 153.48	[352.12, 525.79]	303.13 ± 60.28	[269.03, 337.24]	159.13 ± 50.98	[132.43, 185.84]	114.31 ± 27.114	[100.11, 128.51]
Cmax [µg/mL]	1.07 ± 0.30	[0.90, 1.23]	1.07 ± 0.21	[0.95,1.19]	0.50 ± 0.13	[0.43, 0.57]	0.45 ± 0.11	[0.39, 0.51]
Absorption [µg/mL/h*ppm]	0.013 ± 0	[0.011, 0.014]	0.010 ± 0.002	[0.008, 0.011]	0.011 ± 0	[0.01, 0.012]	0.007 ± 0.0017	[0.006, 0.008]
Physical activity								
AUC [µg*min/mL]	998.64 ± 153.12	[848.58, 1148.70]	529.29 ± 138.25	[393.81, 664.77]	307.99 ± 53.51	[255.55, 360.43]	185.368 ± 40.53	[145.65, 225.09]
Cmax [µg/mL]	2.76 ± 0.26	[2.51,3.02]	1.81 ± 0.47	[1.35,2.27]	1.23 ± 0.09	[1.14, 1.31]	0.71 ± 0.14	[0.57, 0.85]
Absorption [µg/mL/h*ppm]	0.038 ± 0.004	[0.03, 0.04]	0.014 ± 0.003	[0.01, 0.02]	0.024 ± 0.002	[0.021, 0.026]	0.0087 ± 0.0008	[0.008, 0.01]

### Urinary and exhaled breath elimination of PGEE and PGPE at rest as well as with physical activity

**Fig. 4:**
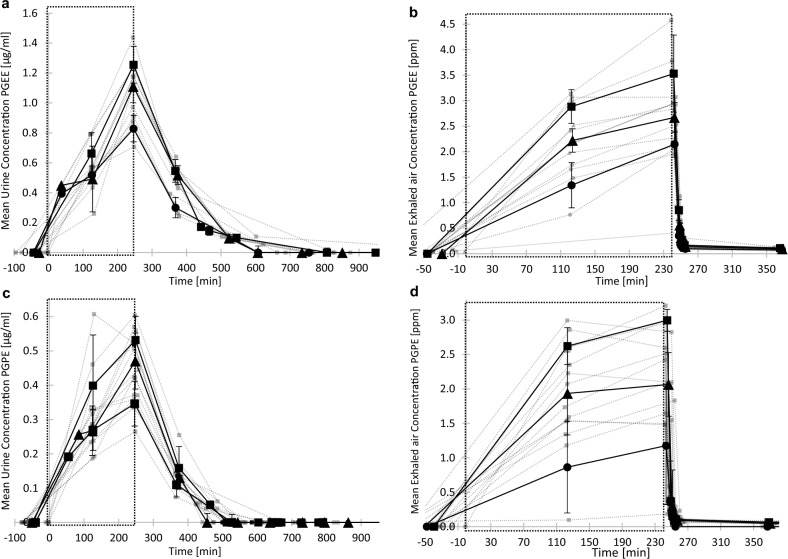
External-internal exposure dose relationship curves in urine (µg/mL) and exhaled breath (ppm) over the time (minutes) following four hours inhalation exposure to PGEE or PGPE at rest. Each grey points represent the data for one participant. Black points are the average (± standard deviation) for all participants (*n* = 5). The large square represents the time in the exposure chamber. Urinary (**a**) or exhaled breath (**c**) PGEE concentrations as a function of time for a 25 (●), 30 (▲) and a 35 (■) ppm exposure to PGEE vapors. Urinary (**b**) or exhaled breath (**d**) PGPE concentrations as a function of time for a 15 (●), 20 (▲) and a 25 (■) ppm exposure to PGPE vapors

The geometric mean of PGEE and PGPE urinary (Fig. 4A and B) and exhaled breath concentrations (Fig. 4C and D) as a function of time for all participants following exposure at rest are represented in Fig. 4. Urine and exhaled breath concentrations of PGEE and PGPE were under the LOQ before exposure for all participants. For both media, the Cmax of PGEE and PGPE in all conditions was reached at the end of exposure. For a same concentration of exposure at rest, the average Cmax in urine were 1.07 ± 0.21 and 0.45 ± 0.11 µg/mL following PGEE and PGPE inhalation exposure respectively. Whereas the average Cmax in exhaled breath were 2.81 ± 0.76 and 2.25 ± 0.66 ppm following PGEE and PGPE inhalation exposure respectively for a same concentration of exposure at rest.

Urinary concentrations were back to below LOQ for all participants 10 h after the end of the exposure sessions at rest. The same results were observed for exposure sessions with physical activity. As in blood, PGEs concentrations in urine and exhaled breath followed a first order (exponential) elimination rate (Figs. 2 and 3). The elimination rates and half-life values were in the same range for all exposure conditions, whatever the air concentration of exposure or the presence/absence of physical activity. Table 2 summarizes the geometric means and the standard deviations as well as the 95% confidence intervals of elimination rates and half-life for both PGEs exposure obtained for all conditions. The urinary half-life for PGEE was 1 h and 20 min and 54 min for PGPE. The elimination rate for exhaled breath was faster with a half-life around 2 min for both solvents.

**Table 2:** Elimination kinetic parameters (geometric mean ± standard deviation) with 95% confidence intervals for PGEE and PGPE in urine and exhaled breath

Parameters	Concentration of solvent			
	PGEE		PGPE	
Urine				
Elimination rate [µg/mL/h*ppm]	− 0.24 ± 0.00	[− 0.26, − 0.22]	− 0.32 ± 0.0602	[− 0.35, − 0.29]
Half-life [h]	1.30	[1.2, 1.4]	0.90	[0.8, 1.0]
Exhaled breath				
Elimination rate [ppm/h*ppm]	− 6.24 ± 0.43	[− 6.703, − 5.768]	− 7.73 ± 0.00	[− 8.235, − 7.231]
Half-life [h]	0.048	[0.052,0.045]	0.038	[0.037,0.042]

## Discussion

A short activity of moderate intensity significantly increases blood absorption of PGEs in the participants exposed by inhalation. The physical activity doubled the internal dose in blood compared to motionless individuals. The internal dose is an important component of the health risk associated with chemical exposure. In most cases, the higher the internal dose, the higher the risk of harmful effects. In the cleaning sector, workers face high physical demands at work. Occupational cleaning is mainly a manual task and is associated with high cardiovascular and muscle load (Søgaard et al. 1996). Thus, during occupational tasks, a higher absorption rate and increased internal dose (AUC) are expected driven by the increased workload (*i.e.*, power expressed in watts [W]) (Johanson 1988; Agneta Löf and Gunnar Johanson 1998). Our results showed also that the blood internal dose increases with air PGE concentrations. However, we did not observe changes in elimination rates or half-lives for both compounds in all media. Therefore, if the internal dose is higher, the organism will take longer to eliminate completely the chemicals. For workers such as painters and cleaners in contact daily approximately eight hours with higher concentrations of PGEs, this means a possible blood accumulation of the substance in the body, and so a greater associated health risk. Although, such occupations require moderate to high physical demands (Eaves et al. 2016; Søgaard et al. 1996), OELs, when they exist, are general not based on studies considering the physical activity and associated physiological changes. Such factors influencing toxicokinetic of chemical substances should be considered in exposure and risk assessments as described by the MAK commission (Hartwig and MAK‐Kommission 2002).

Another important factor to consider in chemicals vapors exposure is the skin absorption. Skin absorption of other organic solvents vapors has been estimated in previous studies between 20 and 75% of the total body intake (Johanson and Boman 1991; Kezic et al. 2000). These percentage differ from those observed with propylene glycol ethers. Brooke et al. (1998) studied the skin absorption of propylene glycol methyl ether (PGME) after exposure to vapors from participants wearing a T-shirt and a pair of shorts, and air-fed masks to exclude inhalation uptake. They estimated that the average dermal contribution to whole-body exposure (compared to inhalation) was 8%, 9.6% and 4.2% of PGME concentration quantified in blood, exhaled breath, and urine respectively (Brooke et al. 1998). This highlights the different toxicokinetic behavior of organic solvents compared with propylene glycol ethers, for which inhalation appears to be a critical route of exposure.

Our results demonstrated a fast absorption into blood (Fig. 2) and a rapid elimination through urine and exhaled breath (Fig. 4), as well as a total urinary elimination of the parent compound after 10 h post-exposure for both PGEs (Fig. 4A and B). These findings confirm results observed in other PGEs toxicokinetic studies performed via the inhalation route (Borgatta et al. 2022; Devanthery et al. 2003; Hopf et al. 2012; Jones et al. 1997). Additionally, we observed a difference in blood absorption between the two solvents. Participants exposed at the same concentration (*i.e.*, 25 ppm) showed a 1.3-fold higher blood concentration following PGEE exposure compared to PGPE (Fig. 2, Table 1). Among important factors that control pulmonary uptake of an inhaled compound, there is the partition coefficient between air and blood (Sato and Nakajima 1987). The greater this partition coefficient, the greater the blood solubility of the compound (Pawson and Forsyth 2008). The partition coefficient blood:air, and so the blood solubility is very high for glycol ethers, *i.e.*, 12′383 for PGME (Johanson and Dynésius 1988). Johanson and Dynésius (1988) determined the blood:air partition coefficient of six different glycol ethers, mostly ethylene glycol ethers. They observed a negative relation between the blood:air partition coefficient and the carbon number (C_X_) of ethylene glycol ethers (Johanson and Dynésius 1988). In this study, we observed a higher blood absorption for PGEE (C4) than for PGPE (C5) for a same concentration of exposure. Based on these observations, we could predict the blood solubility and thus the blood absorption for other commercialized PGEs less studied such as propylene glycol butyl ether (PGBE) or propylene glycol phenyl ether (PGPhE).

Our results demonstrated also that the appropriate blood or urine collection time for PGEs is at the end of the exposure, *i.e.*, directly at the end of the shift for workers. Thus, blood and urinary concentrations are good biomarkers of exposure for PGEE and PGPE. However, obtaining a urine sample is non-invasive and easy to collect. Between 53 and 72% of the total PGEE blood uptake was quantified in urine and less than 0.5% in exhaled breath, while for PGPE, 69.5% of the total blood uptake was quantified in urine and 1.1% in exhaled breath. Our results and those of previous studies show that the quantification of parent compound in exhaled breath is less suitable as an exposure biomarker compared to blood or urinary concentrations. In fact, the half-life is too short to monitor and do not reflect PGEs inhalation exposure (Johanson 1988). Low concentrations of PGEs in alveolar air (Fig. 4C and D) and a rapid post-exposure decrease in exhaled breath was already observed for PGME (Stewart et al. 1970). Other studies accounted exhalation for less than 0.4% of the total uptake after ethylene glycol ethyl ether (EGEE) or its relative acetate (EGEEAc) exposure in humans (Groeseneken et al. 1986, 1987). Groeseneken et al. (1989) observed a high retention of ethylene glycol methyl ether (EGME) in lungs, and a minimal desorption during exhalation. They concluded that a low alveolar concentrations is measured because almost all EGME reaching the alveolar space is directly transferred to the blood (Groeseneken et al. 1989). Thus, a low concentration of glycol ethers in exhaled breath could indicate a high affinity for blood and an absorption already by the bronchial tubes or that solvents remained stuck to the lung walls.

## Conclusion

This study represents the first investigation of PGEE and PGPE human toxicokinetic following inhalation exposure. These chemicals are used between 1000 and 100,000 tons per year in more than 1000 consumer products such as paints, cosmetics, or pharmaceuticals. Despite their widespread use, no toxicokinetic data exist for most of the propylene glycol ethers present on the market. This study contributes to the understanding of human toxicokinetics of common substances never studied before providing an exposure-internal dose relationship for both solvents in resting conditions, a timing of urine and blood collection to assess exposures in future biomonitoring campaigns and biomarkers of exposure for PGEE and PGPE. This study contributes also to the understanding of how physical activity impacts toxicokinetics for these compounds demonstrating that workload should be considered in risk assessments of exposed populations. These finding highlights the need for further investigations for other substances from the same chemical’s family.

## Data Availability

All data have been shown in this publication. If someone wants the exact numbers in table format, this researcher can contact the corresponding author. There is no need for a global statement.
